# Paradoxical Augmentation of Experimental Spondyloarthritis by RORC Inhibition in HLA-B27 Transgenic Rats

**DOI:** 10.3389/fimmu.2021.699987

**Published:** 2021-09-06

**Authors:** Melissa N. van Tok, Mohamed Mandour, Joseph Wahle, Mark E. Labadia, Marleen G. H. van de Sande, Gerald Nabozny, Dominique L. Baeten, Leonie M. van Duivenvoorde

**Affiliations:** ^1^Department of Clinical Immunology and Rheumatology, Amsterdam Rheumatology & Immunology Center (ARC), Amsterdam University Medical Centers (UMC), Location Academic Medical Center, University of Amsterdam, Amsterdam, Netherlands; ^2^Department of Experimental Immunology, Infection and Immunity Institute, Amsterdam University Medical Centers, Location AMC, University of Amsterdam, Amsterdam, Netherlands; ^3^Immunology and Respiratory Diseases, Boehringer Ingelheim Pharmaceuticals Inc., Ridgefield, CT, United States

**Keywords:** spondyloarthritis, IL-17A, RORC, IL-22, HLA-B27 transgenic rats

## Abstract

**Objective:**

IL-17A plays a major role in the pathogenesis of spondyloarthritis (SpA). Here we assessed the impact of inhibition of RAR related orphan receptor-γ (RORC), the key transcription factor controlling IL-17 production, on experimental SpA in HLA-B27 transgenic (tg) rats.

**Methods:**

Experimental SpA was induced by immunization of HLA-B27 tg rats with heat-inactivated *Mycobacterium tuberculosis*. Splenocytes obtained at day 7, 14 and 21 after immunization were restimulated *ex vivo* to assess the induction of pro-inflammatory cytokines. Rats were then prophylactically treated with a RORC inhibitor *versus* vehicle control. The biologic effect of RORC inhibition was assessed by pro-inflammatory cytokine expression in draining lymph nodes. Arthritis and spondylitis were monitored clinically, and the degree of peripheral and axial inflammation, destruction and new bone formation was confirmed by histology.

**Results:**

*Ex vivo* mRNA and protein analyses revealed the rapid and selective induction of IL-17A and IL-22 production by a variety of lymphocyte subsets upon disease induction in HLA-B27 tg rats. Prophylactic RORC inhibition *in vivo* suppressed the expression of IL-17A, IL17F, and IL-22 without affecting the expression of other T helper cell subset related genes. This biological effect did not translate into clinical efficacy as RORC inhibition significantly accelerated the onset of arthritis and spondylitis, and aggravated the clinical severity of arthritis. This worsening of experimental SpA was confirmed by histopathological demonstration of increased inflammation, destruction, and new bone formation.

**Conclusion:**

Despite a significant suppression of the IL-17 axis, RORC inhibitor treatment accelerates and aggravates experimental SpA in the HLA-B27 tg rat model.

## Introduction

The IL-17 axis plays an important role in the immunopathology of spondyloarthritis (SpA) as indicated by a wealth of genetic, immunopathological, experimental, and translational evidence (1). Randomized clinical trials have formally demonstrated the role of IL-17A, the prototypical IL-17 cytokine, in human SpA. Treatment with Secukinumab (anti-IL-17A mAb) reduced signs and symptoms of ankylosing spondylitis (AS) as well as of psoriatic arthritis (PsA), the major forms of axial and peripheral SpA, respectively (2, 3). Similarly, ixekizumab has proven clinical efficacy in PsA (4) and AS (4). Recent evidence, however, suggests that other cytokines of the IL-17A family, with as prime example IL-17F, can contribute to inflammation in SpA (5). Albeit not proven clinically, animal models have also suggested a potential role for IL-22 (6, 7). This raises the question whether targeting other molecules in the IL-17 axis could enhance clinical efficacy above and beyond selective IL-17A blockade.

One of the key molecules controlling the production of IL-17A and related cytokines is the transcription factor retinoic acid receptor-related orphan receptor RORγt (murine) or RORC (the human homologue). RORC is involved in the production and regulation of IL-17A by different cell types including Th17 cells (8), γδ T cells (9), innate lymphoid cells (10). In animal models a distinctive group of RORγt+ Tregs that is vital to maintain gastrointestinal homeostasis and avoid colitis (11–13). Moreover, it is crucial in orchestrating the differentiation of naïve CD4 T cells to Th17 cells (8). Enhanced gene transcription of RORC increases IL-17A production in a T cell line and human primary cells (14–16), whereas blockade of RORC in human CD4 T cells suppresses IL-17A and other inflammatory cytokines, including IL-17F and IL-22 (17, 18). Expression of IL-23R, CCR6, and IL-26 were also decreased upon RORC inhibition in Th17 cells, without affecting the gene signature of other T helper cell types (18, 19). Genetic lack of RORγt protected mice against experimental autoimmune encephalomyelitis (EAE), induced defects in Th17 differentiation and prevented T-cell-transfer-mediated colitis (8, 20). Pre-clinical studies in animal models, including imiquimod-induced psoriasis (21), spontaneous colonic inflammation (22), antigen induced arthritis (17), EAE (23), and experimental autoimmune uveitis (EAU) (24), confirmed that RORC inhibition markedly reduces local and systemic IL-17A levels and decreases tissue inflammation. Moreover, expression of IL-17F and IL-22 was also reduced upon *in vivo* RORC inhibition (21, 23). These findings indicate that RORC could be an interesting therapeutic target in IL-17A dependent pathology, blocking not only IL-17A but also related pro-inflammatory cytokines.

In this study, we aimed to assess RORC as potential therapeutic target in SpA by studying the effects of a small molecule RORC inhibitor in experimental SpA in HLA-B27 transgenic (tg) rats, a well validated model for human SpA (25–27). As we previously showed that both initiation and disease persistence in this model is partially but not completely inhibited by IL-17A blockade (28), we used this model to assess how RORC inhibition affects a panel of IL-17 related cytokines *in vivo* and if this biological effect translates into clinical efficacy.

## Methods

### Animals

The inducible HLA-B27 tg x beta-2 microglobulin (B2M) tg rat model has been described previously (27). Briefly, the Tg (HLA-B*2705,B2M) 21-3Reh and Tg(B2M) 283-2Reh rat lines (25) on Lewis background were bred and housed (3 per cage) in individually ventilated cages at the animal research institute AMC. Six weeks-old F1[21-3x283-2] animals were immunized with low dose (30-90 µg depending on sex and housing conditions) heat-inactivated *Mycobacterium tuberculosis* (MTB) (Difco, Detroit, MI) in 100 µl Incomplete Freund’s Adjuvant (IFA) (Chondrex) *via* subcutaneous injection in the tail base. The immunization strategy using heat-inactivated *Mycobacterium tuberculosis* as a broad innate immune receptor trigger. In the HLA-B27 tg rats, immunization using 30–60 µg *M. tuberculosis* was sufficient to induce spondylitis and arthritis in both male and female rats (80-100%). Without immunization spontaneous development of spondylitis and arthritis appeared in 40% of the male rats around 9 months of age in the presence of a severe autoimmune epididymoorchitis which is clinically manifested in 100% of the male rats at 3 months of age. Clinical disease (arthritis and spondylitis) appears 20-30 days after immunization. All animal experiments were approved by the AMC Animal Care and Use Committee, in line with national and international regulations and guidelines. The data presented are the data of two separated experiments combined.

### *Ex Vivo* Restimulation Experiments

Mononuclear cells from spleen were isolated from F1[21-3x283-2] rats at day 7, 14, 21 after immunization with MTB in IFA (n=4/time point). At each time point two non-immunized rats matched by age and gender, were taken along as control. The non-immunized rats (n=6) were pooled for all analyses. Cells were used either unstimulated or after *ex vivo* restimulation with 10 ng/ml PMA and 1 µg/ml ionomycin for 6 hours. Using splenocytes, mRNA expression was measured using Taqman assays (Thermo Fisher Scientific) for *il17a* (assay ID Rn01757168_m1), *tnf* (assay ID Rn99999017_m1) and *ifng* (assay ID Rn00594078_m1) with *gapdh* (assay ID Rn01775763_g1) as housekeeping gene. Data were analyzed according to the 2^-ΔΔCT^ method (29). IL-17A, TNF, and IFNy protein secretion was measured by ELISAs in the culture supernatant stimulated for 24 hours (Thermo Fisher Scientific IL-17A ELISA kit #88-7170-88; R&D duoset IFNy ELISA #DY-585). Intracellular protein expression of IL-17A and IFNy (*versus* isotype control) by different lymphocyte subsets was assessed by FACS in splenocytes stimulated overnight (in the presence of 10 µg/ml Brefeldin for the final 4 hours). Data was recorded using a FACS Canto II and analyzed using FlowJo software.

### *In Vivo* RORC Inhibition

The RORC inhibitor BI119 (Boehringer-Ingelheim Pharmaceuticals Inc., Ridgefield, CT, USA) was discovered by screening a small-molecule compound library. BI119 strongly bound to the human RORyt ligand-binding domain (LBD) and was active in an RORyt LBD reporter assay (Kd for RORy LBD – 65 nM; IC50 for RORy LBD reporter assay 260 nM). The compound showed high selectivity towards RORyt as demonstrated by a lack of significant activity against RORa (IC50 > 10 μM) and RORb (IC50 > 6 μM). Rats (n=6/group) received 30 mg/kg RORC inhibitor dissolved in Natrosol™ 250 Hydroxyethylcellulose (further referred to as Natrosol) (Ashland Specialty Ingredients #88-7170-88) or Natrosol alone twice daily *via* oral gavage. Assignment to treatment was random and per cage to avoid cross contamination *via* feces. Treatment started one week after immunization and continued for five weeks. Experiment was done twice with 6 rats/group.

### Serum Exposure Measurement

Serum samples were collected *via* Saphenous vein puncture, 2 hours after the morning dose at day 31 and before the morning dose at day 32 post immunization. The concentration of BI114 was determined by liquid chromatography-mass spectrometry analysis.

### Downstream Cytokine Analyses

After *in vivo* treatment, RNA was isolated from popliteal lymph nodes using TRIzol. Samples were analyzed with a rat Th17 qPCR array according to the manufacturers protocol (Qiagen # PARN-073Z). Selective genes were confirmed by regular qPCR with SYBR green primers for IL-17A, IL-22, IL-17F, IL-13 and GAPDH as housekeeping gene (all primer sequences are available upon request). Data were analyzed according to the 2^-ΔΔCT^ method (29).

### Clinical Scoring of Arthritis and Spondylitis

The presence of arthritis in the paws was determined clinically and digital hind paw swelling was measured with plethysmometry. Arthritis severity in each paw was graded 0-3 as described before (27). Cumulative clinical scores were calculated for severity analysis. Swelling in cm^3^ was normalized to the days before disease onset. Spondylitis was determined clinically by swelling and bumps in the tail and scored yes/no. In case of humane endpoints, due to ethical considerations, rats were sacrificed with the last observation carried forward. Humane endpoints were defined as 15% bodyweight loss or two completely swollen paws. One rat in the vehicle treated group was sacrificed due to reaching the humane endpoint for bodyweight loss. Clinical scoring was performed by one observer, blinded for treatment.

### Histology

Hind paws and tails were decalcified in Osteosoft (Merck) and embedded in paraffin. Sections were stained for hematoxylin and eosin or safranin O and semi-quantitatively scored by two observers blinded for treatment (MT, LD) as previously described (26).

### Statistics

Data were analyzed using GraphPad prism 7 software. Spondylitis and arthritis incidence were analyzed using a survival curve. Comparison of survival curves was analyzed using the Log-Rank (Mantel-Cox) test. Arthritis severity (arthritis score and hind paw swelling) was analyzed using the area under the curve followed by a Mann-Whitney U test. All other data were analyzed using a Mann-Whitney U test.

## Results

### Induction of Experimental SpA in HLA-B27 Tg Rats Is Associated With a Rapid and Selective Induction of IL-17A and IL-22

**Figure 1:**
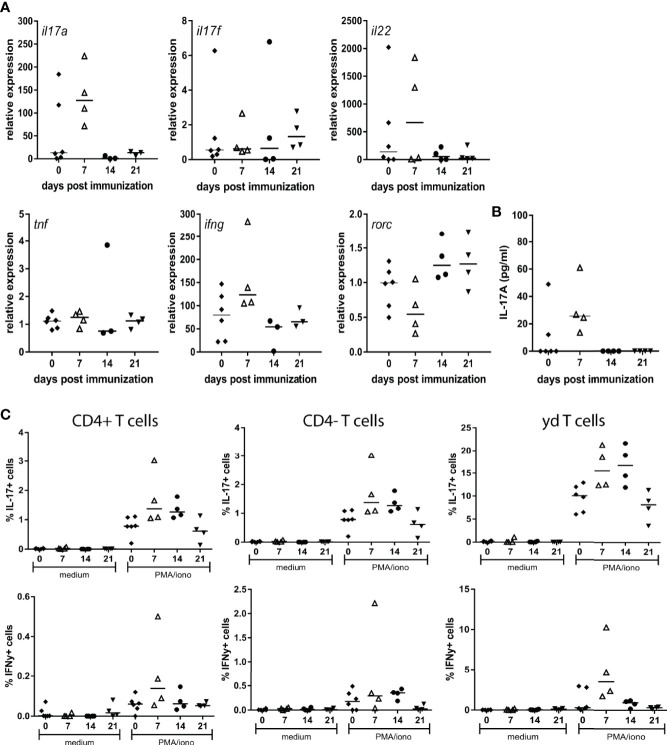
Restimulation of splenocytes from MTB immunized HLA-B27 tg rats primarily induced IL-17A. **(A)** Relative expression of il17a, il17f, il22, tnf, ifng and rorc **(B)** protein secretion of IL-17A **(C)** FACS analysis of IL-17A and IFNy expressing T cell subsets.

We previously demonstrated that selective blockade of IL-17A significantly reduces spondylitis and arthritis development in the inducible HLA-B27 tg rat model of SpA (28). To further assess the potential involvement of IL-17A and related pro-inflammatory cytokines, *ex vivo* cellular responses to MTB immunization were assessed. To this end cytokine expression and production were determined in splenocytes upon restimulation with PMA/Ionomycin at 7, 14 and 21 days after MTB immunization. RNA expression analysis indicated a trend towards an increase in IL-17A, but not in IL-17F expression upon restimulation, with the peak-response at 7 days after immunization. Furthermore IL-22 was increased upon restimulation at 7 days after immunization. Other pro-inflammatory cytokines including TNF and IFNy as well as the expression of RORC, were not changed after immunization (**Figure 1A**). Protein secretion analysis confirmed the trend towards increased levels of IL-17A at 7 days after immunization (**Figure 1B**), while secretion of TNF and IFNy was not detectable at any time point upon restimulation with PMA/Ionomycin. A repetitive experiment with samples collected at day 7 (n=2 non-immunized and n=3 immunized), confirmed the increase in IL-17A expression, and the unchanged expression of IFNy upon immunization. To assess which cells were responsible for IL-17A production in our model FACS analysis was performed, focusing on three T cell subsets: CD4+, CD4- and γδ T cells. The increased presence of IL-17A^+^ cells at 7 days after immunization could be confirmed, within the population of CD4+TCRab^+^, CD4-TCRab^+^ and CD4-TCR γδ^+^ T cells (**Figure 1C**). The frequency of IFNy^+^ cells was low in all subsets, with no differences between the different time points (**Figure 1C**). Collectively, these data indicate the rapid and selective induction of IL-17A production by a variety T cell subsets upon MTB immunization in HLA-B27 tg rats.

### *In Vivo* RORC Inhibition Suppresses IL-17A, IL-17F and IL-22 Expression in HLA-B27 Tg Rats

**Figure 2:**
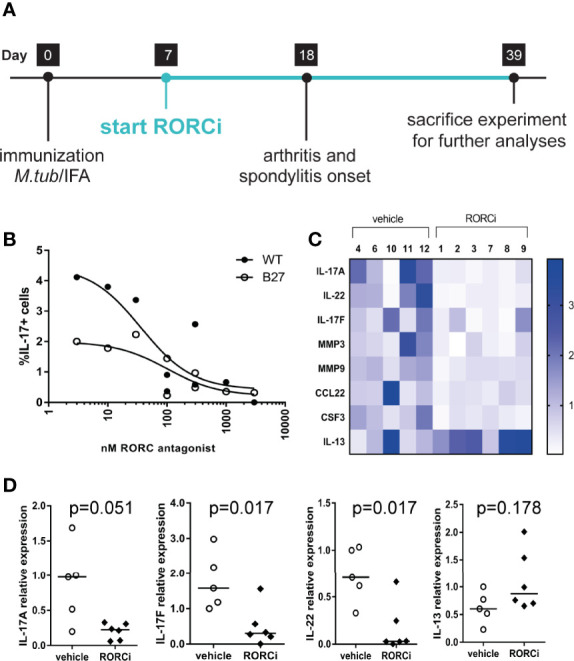
*In vivo* and *in vitro* inhibition of RORC. **(A)** Study design of *in vivo* prophylactic treatment **(B)** Dose response effect of *in vitro* RORC inhibition on frequencies of IL-17+ yd T cells. **(C)** Relative expression of genes >2fold differentially regulated in vehicle *vs* RORCi treated rats. **(D)** Gene expression from the selected genes, IL-17A, IL-22, IL-17F and IL-13 was confirmed by regular qPCR. Data are individual data points and median.

To assess the potential relevance of RORC - the key transcriptional regulator of IL-17A and related cytokines - as therapeutic target, we performed an *in vivo* prophylactic treatment study with RORC inhibition *versus* vehicle control in our HLA-B27 tg at model (**Figure 2A**). Serum measurements confirmed that all rats in the treatment group, but not in the vehicle control group, had high exposure to the compound (**Table 1**). Whereas a variety of previous experiments with human/mouse cells indicated an IC50 of 10 to 20 nM (data not shown), we confirmed that the exposure levels seen *in vivo* in the rats significantly inhibited IL-17A production by γδ T cells of both wildtype and HLA-B27 tg rats *in vitro* (**Figure 2B**).

**Table 1:** Serum levels of RORC inhibitor.

Rat	Treatment	Concentration (nM)	
		before	after
1	RORCi	2491	2055
2	RORCi	1487	1576
3	RORCi	1425	1873
4	Vehicle	0	0
5	Vehicle	0	0
6	Vehicle	0	0
7	RORCi	3527	4691
8	RORCi	1187	4673
9	RORCi	1891	1762
10	Vehicle	0	0
11	Vehicle	0	0
12	Vehicle	0	0

RORCi serum exposure was measured in rats of both groups (RORCi vs vehicle) before the morning dose and 2-3 hours after the morning dose at 1 timepoint only: day 32/33 of treatment.

We next investigated whether the expression of IL-17A and related cytokines was also reduced *in vivo* by RORC inhibition by analyzing popliteal lymph nodes obtained at the end of the *in vivo* study. IL-17A, IL-17F, and IL-22 expression was more than 2-fold downregulated as measured in a qPCR array (**Figure 2C**). These data were confirmed by regular qPCR (**Figure 2D**). The Th2 cytokine IL-13 initially showed a tendency towards an increase upon RORC inhibition in the arrays (**Figure 2C**) but this could not be confirmed by regular qPCR (**Figure 2D**). Expression of a wide panel of Th1, Th2, and Treg cytokines or transcription factors, or other genes included in the Th17 array, was not different between vehicle treated and RORCi treated rats. Together these data indicate that RORC inhibition *in vivo* suppressed the expression of IL-17A, IL17F, and IL-22 without affecting the expression of other T helper cell subset related genes.

### *In Vivo* RORC Inhibition Augments Experimental SpA in HLA-B27 Tg Rats

**Figure 3:**
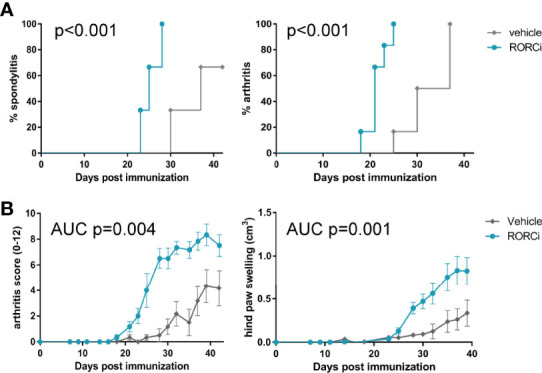
RORC antagonist accelerated spondylitis and arthritis development and increased arthritis severity. **(A)** Incidence of spondylitis and arthritis in vehicle *vs* RORCi treated rats **(B)** disease severity, displayed as arthritis score and hind paw swelling in vehicle *vs* RORCi treated rats. Data are mean +/- SEM.

**Figure 4:**
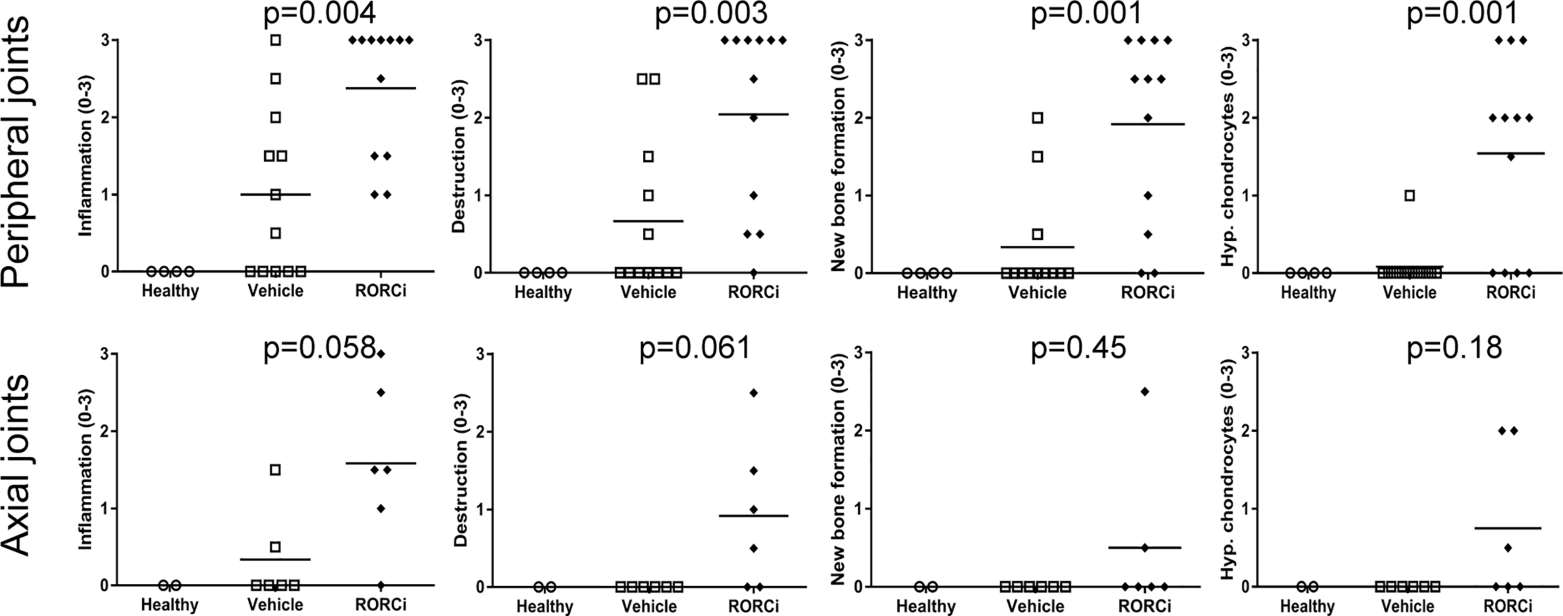
Increased histological pathology in RORC inhibitor treated rats. Peripheral and axial joints were semi-quantitatively scored (0–3) for inflammation, destruction, new bone formation and hypertrophic chondrocytes.

To assess if the observed biological effects on the IL-17 axis translate into clinical efficacy, clinical disease development was monitored over time and histopathological analysis was performed at the end of the study. All RORC inhibitor-treated rats developed both spondylitis and arthritis *versus* 70% and 100%, respectively, in the vehicle-treated group (**Figure 3A**). The mean onset of spondylitis was day 25 in RORC inhibitor *versus* day 34 in vehicle treated rats. The mean onset of arthritis was day 21 in RORC inhibitor *vs* day 33 in vehicle treated rats. In terms of arthritis severity, both arthritis score (p=0.004) and hind paw swelling as assessed by plethysmometry (p=0.001) were increased upon RORC inhibition (**Figure 3B**). Histopathological analysis of the peripheral joints at the end of the experiment confirmed a significant increase in inflammatory infiltration (p=0.004), destruction (p=0.003), newly formed bone (p=0.001), and hypertrophic chondrocytes (p=0.001) in RORC inhibitor treated rats compared to the controls (**Figure 4**). A similar trend towards increased inflammation (p=0.058) and bone destruction (p=0.061) was observed in the spine (**Figure 4**). Collectively, these data consistently demonstrate that, despite the expected biological impact of RORC inhibition on the IL-17 axis, this treatment did not inhibit but rather accelerated and aggravated experimental SpA in HLA-B27 tg rats. To investigate if RORgt inhibition affected the gut we performed histology on the colon and small intestine. No inflammation was observed in the colon and the small intestine. Combined with the absence of weight loss, these data show that the aggravation of the SpA phenotype cannot be explained by triggering of disease by RORgt inhibition induced gut inflammation.

## Discussion

The major findings of the current study are 1) that IL-17A is primarily produced upon restimulation of splenocytes from MTB immunized rats, with a peak response after 7 days of immunization. 2) IL-17A expression could be reduced upon *in vitro* as well as *in vivo* RORC inhibition, additionally *in vivo* RORC inhibition reduced IL-17F and IL-22 expression without affecting other T helper cell related genes. 3) Despite these molecular changes RORC inhibition did not inhibit, but rather stimulated disease development. These findings are surprising considering previous studies of RORC inhibition in a variety of inflammatory models including antigen-induced arthritis (17), imiquimod-induced psoriasis (21), IL-23 induced skin inflammation (30), intestinal inflammation (22, 31). Recently, Tan et al. (24) showed the reduced clinical severity of experimental autoimmune uveitis (EAU), and EAE using two different RORc antagonists (CQMU151 and CQMU152) (24).

All these models showed decreased levels of IL-17A and significant reduction of clinical symptoms upon pharmacological RORC inhibition or in RORC deficient animals. Also in 2018, Taurog et al. demonstrated the therapeutic efficicacy of the RORγt antagonist (A-1619758) in reducing inflammation, and suppression of both axial and peripheral skeletal bone changes in the HLA-B27 transgenic rat deficient for *Dazl gene* (21-3x283-2x17-9) (32).

A major question is whether the data of the current study could have been biased by a technical or biological artefact. Exposure measurements indicated that all rats in the treatment group have high levels of RORC inhibitor present in their serum shortly before and a few hours after treatment. The control group responded as expected, although disease incidence and severity were low. We could detect a biological effect, in terms of reduced IL-17A, IL-17F and IL-22 expression and finally clinical data are in line with histological data. Together these findings prove that groups were not switched during or after this experiment. The potential mechanism remains unknown.

**Figure 5:**
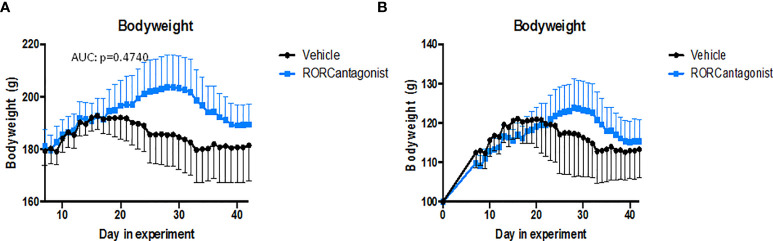
Body weight follow up in treated rats. **(A)** experiment 1. **(B)** experiment 2.

Others showed, using the exact same RORC inhibitor, that while IL-17A levels were reduced, the production of IL-22 continued (33, 34) and treatment with the RORC inhibitor abrogated experimental colitis (34). Specific subsets of iNKT and γδ T cells showed to be among the IL-22 producing cells upon RORC inhibition (33). Similarly another RORC inhibiting compound could selectively impact specific cell types, including Th17 cells, while local IL-17A or IL-22 production by ILC3s was not reduced in a mouse model for intestinal inflammation (22). IL-22 has been shown to be a key player in intestinal host defence and mucosal homeostasis (35), and as we did observe reduced IL-22 expression, it could by hypothesized that RORgt inhibition could result in gut inflammation by a decrease in IL-22. This gut inflammation could then induce the SpA phenotype in the rats, similar to the induction of disease by orchitis, which is a consistent finding in this animal model. However, the rats showed no weight loss (**Figures 5A, B**) and gut inflammation was absent after RORgt inhibition, which makes this hypothesis very unlikely.

In contrast, in another model for intestinal inflammation the RORgt inverse agonist (TAK-828F) suppressed intestinal inflammation. In this model the compound significantly reduced Th17 and Th17/Th1 cell population in mesenteric lymph nodes (MLN) which was accompanied by suppressed/decreased gene expression of not only IL17A, IL17F but also IL-22. Local effects on ILC3 function in the intestine was not studied. However, in normal mice this RORgt inverse agonist did reduce numbers of Th17 cells and IL3s in the laminal propria (31).

In order to test the responsiveness of cells in the MTB immunized HLA-B27 tg rat CD4^+^ T cells and γδ T cells were isolated from spleen and draining lymph nodes and restimulated in the presence of RORC inhibitor (n=9). IL-17A and IL-22 expression were similarly decreased in both cell subsets upon RORC inhibition.

It is important to note, however, that this is not the first time that clinical or preclinical data demonstrate that the IL-17 axis does not work in a linear manner. It was reported by Chong et al., that loss of IL-17A produced by Th17 cells didn’t reduce the pathogenicity of these cells as expected and instead increased the expression of other Th17 cytokines (i.e. GM-CSF and IL-17F) (36). This was attributed to a Th17 cell-intrinsic autocrine loop induced by IL-17A binding to its receptor resulting in IL-24 induction, which in turn repressed the Th17 cytokine program. They showed that *in vivo* IL-24 treatment ameliorated Th17-induced EAU, whereas silencing of IL-24 in Th17 cells enhanced disease. However, these findings are unlikely to explain the paradoxical effects of RORC inhibition in our model as we observed the down regulation of other TH17 cytokines (e.g. IL-17F).

Clinical studies in psoriasis patients indicated that both blockade of IL-23 as well as IL-17A are effective (37, 38). However in Crohns disease blockade of IL-23 was effective while blockade of IL-17A or IL-17RA was not (39–41). Finally in SpA, blockade of IL-23p19 seems to be effective in psoriatic arthritis (42) but not in ankylosing spondylitis (43). Similarly in a previous study with our HLA-B27 tg rats, we showed that blockade of the IL-23 receptor (IL-23R) significantly reduced levels of IL-17A and IL-22 (but not IL-17F) *in vivo*. While prophylactic blockade of the IL-23R completely prevented spondylitis and arthritis development, therapeutic blockade did not impact clinical or histological spondyloarthritis in the HLA-B27 tg rat (44). Moreover prophylactic as well as therapeutic blockade of IL-17A was effective in this model (28).

The RORγt+ Tregs subset might be involved in these unanticipated effects. The function of these regulatory cells was thought to be controlled partly by RORγt (45). Kleinewietfeld et al. showed that a low molecular weight RORγt inhibitor could influence the frequencies of regulatory T-cells (Tregs) beside the Th17 response. The equilibrium between RORγt and FoxP3 expression level would equally control Treg and Th17 cells, depending on the cytokine environment. RORγt inhibition would twist the Th17/Treg cell ratio towards the Treg pathway (46). Despite the apparent beneficial effect for inducing immune tolerance, it seems that RORγt modulation would alter both the downstream pro- and anti-inflammatory pathways and induce undesirable effects (45). Affecting the regulatory function of these RORγt Tregs would predispose to inflammation (even when the expression of Th17 signature genes and cytokines is decreased). The question remains to what extend these findings are relevant for other models or for human disease. Based on our previous findings regarding IL-23R and IL-17A blockade in the HLA-B27 tg rats we conclude that it is uncertain whether RORC inhibition might be a good therapeutic option for all IL-17A driven diseases.

## Data Availability Statement

The original contributions presented in the study are included in the article/supplementary material. Further inquiries can be directed to the corresponding author.

## Ethics Statement

All animal experiments were approved by the AMC Animal Care and Use Committee, in line with national and international regulations and guidelines.

## Author Contributions

MT and LD, study design, experimental procedures, analyzing data, writing manuscript. MM, analyzing data, writing manuscript. JW, experimental procedures. ML, study design, experimental procedures, analyzing data. MS, analyzing data, writing manuscript. GN and DB, study design, analyzing data, writing manuscript. All authors contributed to the article and approved the submitted version.

## Funding

This work was sponsored by Boehringer-Ingelheim Pharmaceuticals Inc., Ridgefield, CT, USA.

## Conflict of Interest

ML and GN are employees of Boehringer-Ingelheim Pharmaceuticals Inc. MS received consultancy fees from Novartis and Abbvie and research grants from Janssen, Novartis and Eli Lilly. DB is an employee of UCB.

The authors declare that this study received funding from Boehringer-Ingelheim Pharmaceuticals Inc. The funder shared in study design, data analysis and data collection.

The remaining authors declare that the research was conducted in the absence of any commercial or financial relationships that could be construed as a potential conflict of interest.

## Publisher’s Note

All claims expressed in this article are solely those of the authors and do not necessarily represent those of their affiliated organizations, or those of the publisher, the editors and the reviewers. Any product that may be evaluated in this article, or claim that may be made by its manufacturer, is not guaranteed or endorsed by the publisher.
